# Survey about do-it-yourself closed loop systems in the treatment of diabetes in Germany

**DOI:** 10.1371/journal.pone.0243465

**Published:** 2020-12-17

**Authors:** Anna Laura Herzog, Jonas Busch, Christoph Wanner, Holger K. von Jouanne-Diedrich

**Affiliations:** 1 Division of Nephrology, Transplantationszentrum, University of Würzburg, Universitätsklinikum, Würzburg, Germany; 2 TH Aschaffenburg (University of Applied Sciences), Aschaffenburg, Germany; 3 Division of Nephrology, Medizinische Klinik I, University of Würzburg, Universitätsklinikum, Würzburg, Germany; 4 Competence Center for Artificial Intelligence, Faculty of Engineering, TH Aschaffenburg (University of Applied Sciences), Aschaffenburg, Germany; University of Salamanca, SPAIN

## Abstract

Continuous glucose monitoring (CGM) improves treatment with lower blood glucose levels and less patient effort. In combination with continuous insulin application, glycemic control improves and hypoglycemic episodes should decrease. Direct feedback of CGM to continuous subcutaneous insulin application, using an algorithm is called a closed-loop (CL) artificial pancreas system. Commercial devices stop insulin application by predicting hypoglycemic blood glucose levels through direct interaction between the sensor and pump. The prediction is usually made for about 30 minutes and insulin delivery is restarted at the previous level if a rise in blood glucose is predicted within the next 30 minutes (hybrid closed loop system, HCL this is known as a predictive low glucose suspend system (PLGS)). In a fully CL system, sensor and pump communicate permanently with each other. Hybrid closed-loop (HCL) systems, which require the user to estimate the meal size and provide a meal insulin basis, are commercially available in Germany at the moment. These systems result in fewer hyperglycemic and hypoglycemic episodes with improved glucose control. Open source initiatives have provided support by building do-it-yourself CL (DIYCL) devices for automated insulin application since 2014, and are used by a tech-savvy subgroup of patients. The first commercial hybrid CL system has been available in Germany since September 2019. We surveyed 1054 patients to determine which devices are currently used, which features would be in demand by potential users, and the benefits of DIYCL systems. 9.7% of these used a DIYCL system, while 50% would most likely trust these systems but more than 85% of the patients would use a commercial closed loop system, if available. The DIYCL users had a better glucose control regarding their time in range (TIR) and glycated hemoglobin (HbA1c).

## Introduction

More than 20 million people worldwide are affected by diabetes mellitus, and 0.5% of the population in Germany suffers from type 1 diabetes mellitus (DMT1) according to the Deutsche Diabetes Gesellschaft (DDG) [1]. The complications of poor glycemic control, such as micro- and macro-angiopathy with infectious complications, major cardiac adverse events (MACEs), and progressive kidney disease, are life-threatening and drastically reduce quality of life [2–4]. The management of complications also increases medical costs for society [5]. The Diabetes Complications and Control Trials have shown that a tighter control of blood glucose with lower HbA1c reduces the risk of these complications [6].

Continuous glucose monitoring (CGM) in combination with rapid-acting insulin analogs have improved glucose control, reducing hypoglycemia and episodes of diabetic ketoacidosis (DKA), resulting in more time spent in optimum glucose levels compared to standard monitoring [7]. In combination with continuous subcutaneous insulin infusion (CSII), glucose control is improved and patient effort decreased as therapy intensification is achieved. A closed loop (CL) system consists of an insulin pump, such as the Ypsomed YpsoPump, the DANA Diabecare^®^ RS, or the Insulet Omnipod^®^, and a corresponding CGM, such as Abbott FreeStyle Libre and Libre 2, Medtronic Guardian^™^ Sensor 3, or Dexcom G6^®^. Insulin pumps can be conventional with a cannula and catheter tube, or patched with an integrated cannula [8]. CGM can be either subcutaneously stitched or implanted [9]. The control algorithm is a type of logic programmed into a controller to analyze the delta between the measured glucose value and a set point, and can either be integrated into the insulin pump or an external device, such as a smart phone, that communicates with both wirelessly. The biggest challenge is to rebuild a fast and sensitive in vivo response system with currently available sensor technology [10, 11]. A hybrid closed loop system (HCL) uses an algorithm capable of automatically adjusting basal insulin delivery in response to CGM readings transmitted to the insulin pump every 5 minutes. Currently available devices are based on sensor-augmented pump systems (SAPs), such as Medtronic’s MiniMed^™^ 640G insulin pump with SmartGuard^™^ that stops insulin application by predicting upcoming hypoglycemic episodes for the subsequent 30 minutes. These systems are not able to increase insulin infusion in response to elevated serum glucose, but reduce hypoglycemic episodes without causing rebound hyperglycemia [12]. The patient’s intervention is still required with self-monitoring of blood glucose levels twice daily for calibration and by considering nutritional intake [13].

More sophisticated controller algorithms, such as proportional–integral–derivative (PID) control, model predictive control, fuzzy logic (FL) control, and adaptive control algorithms have been developed and extensively evaluated for insulin delivery in AP systems [14, 15].

HCL systems require manual meal boluses and are preferred, as the algorithms in full CL systems require adaptation for compensation of post-prandial hyperglycemias, the first system was FDA approved for home use in 2016 and has been available in Germany since September 2019 [16, 17].

With the lack of an available system until the last year, Dana Lewis and Scott Leibrand started an open source project in 2014 to provide the medical systems to every patient and force the development of CL systems [18]. This already shows that there is definitely a need for such systems. The OpenAPS project is the first project for a do-it-yourself CL (DIYCL) system. An insulin pump (from a defined insulin pump group), a CGM (from a defined CGM group), a system-on-chip or “single board computer” (Intel Edison or Raspberry Pi with installation), an attachment (connections, radio transmitter, etc.), and battery are required. This electronic device then communicates with the insulin pump and CGM to perform actions, such as the application of insulin or issuing warnings via a smartphone for hypoglycemia or hyperglycemia [19].

The Android APS project is similar to the OpenAPS project and is based on the same algorithm. Android APS also features functions super micro bolus (SMB) and unannounced meals (UAM). The big difference is that this variant uses an Android smartphone for the algorithm instead of the self-built electronics. Communication between the insulin pump and CGM is via Bluetooth^™^, and the selection of possible devices changes. To use all functions, different objects must be reached, which requires that the user has spent some time on the subject [20].

Unlike AndroidAPS, Loop is based on an iPhone. For the communication between the pump and smartphone, additional electronics are required to establish a radio connection. The function is very similar to the other two DIYCL systems, as an algorithm analyzes the data provided by the insulin pump and CGM and makes decisions as to how to deliver insulin in the case of hyperglycemia. Nevertheless, there are differences from other systems; for example, the UAM functions are not available [21].

All three DIYCL systems use Nightscout as their documentation platform [22].

The algorithm in a CL system calculates a future glucose value from various input data at a certain time and derives decisions from this to correct the current glucose value to the target glucose value. This is mostly achieved by adjusting the basal rate. Depending on the active insulin in the body and the future glucose value, further decisions are made regarding adjustment of the temporary basal rate. This routine is performed at regular repetitions in single-digit low-minute intervals. The new glucose value measured by the CGM or the data from the insulin pump (e.g., IOB net) are used for decisions.

In the present study, we created a survey to determine the satisfaction of patients with the current products available on the market and the use and requirements of DIYCL and commercial CL systems.

## Methods

The target group for the online survey was patients with Diabetes mellitus who use an insulin pump, an insulin pen, a continuous glucose meter, and/or a blood glucose meter at the time of the survey. The patient demographics are given in Table 1. Informed consent was waived by the ethics commitee of the university of Wuerzburg due to anonymous data collection (20200618 01). Since the prerequisite for participation in the survey or for answering the questions is the use of these tools, and since the survey is primarily aimed at user satisfaction, application behaviour and suggestions for improvement, 12 patients with T2DM (1,1%) were not excluded. The questionnaire was based on the comments of Jacob et al. and Porst [23, 24]. To share the survey link, a publishing house (Diabetes Journal) and the largest online communities linked to patients with DMT1 were contacted. General questions comprised demographic parameters, such as age, type and duration of diabetes, devices used, place of residence, and the use of a DIYCL. Another part contained questions about the type of pump or CGM, contentment and criticism, and which application form was preferred. The questionnaire about CL systems asked for the applied system, the time in range, hypoglycemic episodes, changes in HbA1c, and contentment and criticism. The last part asked about future therapies using a DIYCL with explanatory statements, trust in future systems, and requirements.

**Table 1:** Patient demographics.

**Gender**	**Male**	297	28.2%
	**Female**	626	59.4%
	**Unknown**	131	12.4%
	**n**	1054	100%
**Age (y)**	**No answer**	126	11.9%
	**0–9**	66	6.2%
	**10–19**	100	9.4%
	**20–29**	149	14.1%
	**30–39**	218	20.6%
	**40–49**	207	19.6%
	**50–59**	138	13.0%
	**60–69**	43	4.0%
	**> 70**	7	0.6%
	**n**	1054	100%
**DM Type**	**1**	1027	97.4%
	**2**	12	1.1%
	**3**	9	0.8%
	**Other**	6	0.5%
**HbA1c (%)**		6.4 ± 3.4	
**Time in range (%)**		64.4 ± 34.7	
	**No answer**	182	
**Length of insulin use (y)**	**< 2**	135	12.8%
	**2–4.9**	168	15.9%
	**5–9.9**	154	14.6%
	**10–19**	236	22.3%
	**20–29**	185	17.5%
	**30–40**	111	10.5%
	**40–50**	57	5.41%
	**> 60**	1	0.09%
	**n**	1054	
**DIYCL**	**all**	102	9.7%
	**AndroidAPS**	79	77.2%
	**Loop for iOS**	4	4.0%
	**OpenAPS**	3	3.0%
**Insulin pump**	**No answer**	353	33.4%
	**AccuCheck**	157	14.8%
	**Animas Vibe**	5	0.4%
	**DANA Diabecare R**	60	5.6%
	**Insulet Omnipod**	98	9.2%
	**MiniMed**	353	33.4%
	**Ypsopump**	28	2.6%
	**n**	1054	
**CGM**	**No answer**	195	18.5%
	**Dexcom**	250	23.7%
	**Eversense**	12	1.1%
	**Freestyle Libre**	382	36.2%
	**Medtronic**	215	20.3%
	**n**	1054	

CGM continuous glucose monitoring, DIYCL Do it yourself closed loop, IDDM insulin dependent diabetes mellitus, DM Type diabetes mellitus, y year.

We used the R environment for statistical computing for statistical analyses and creation of the plots [25]. For the correlation of the HbA 1c value to the TIR the Shapiro-Wilk test on normal distribution was used. Based on this, the Spearman correlation coefficient was used, resulting in a correlation coefficient of -0.542 (significant at significance level 1%). In addition, it was investigated whether users of a DIYCL system have a lower HbA 1c value compared to non-users. To this end, a Levene-test was performed to check the equality of variance and then a t-test was performed for independent samples.

## Results

### Therapeutic usage

**Fig 1:**
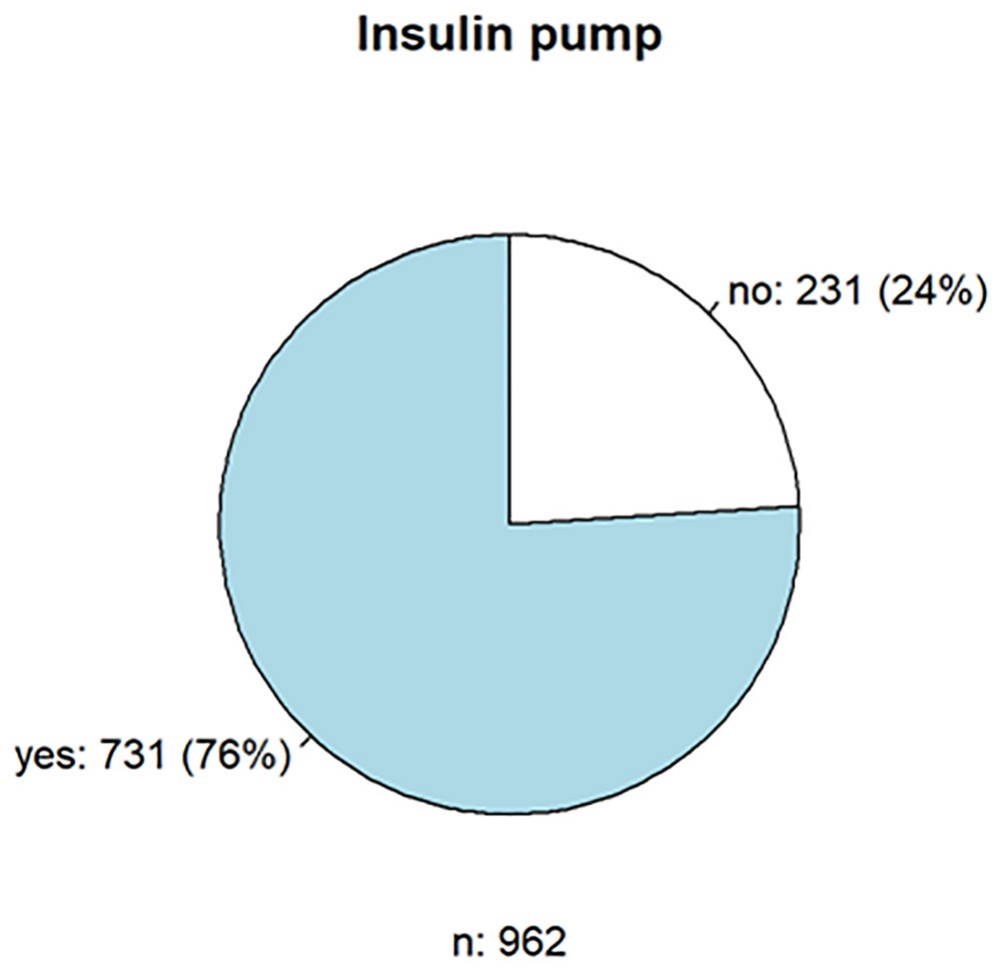
Frequency of insulin pump use in diabetes therapy.

**Fig 2:**
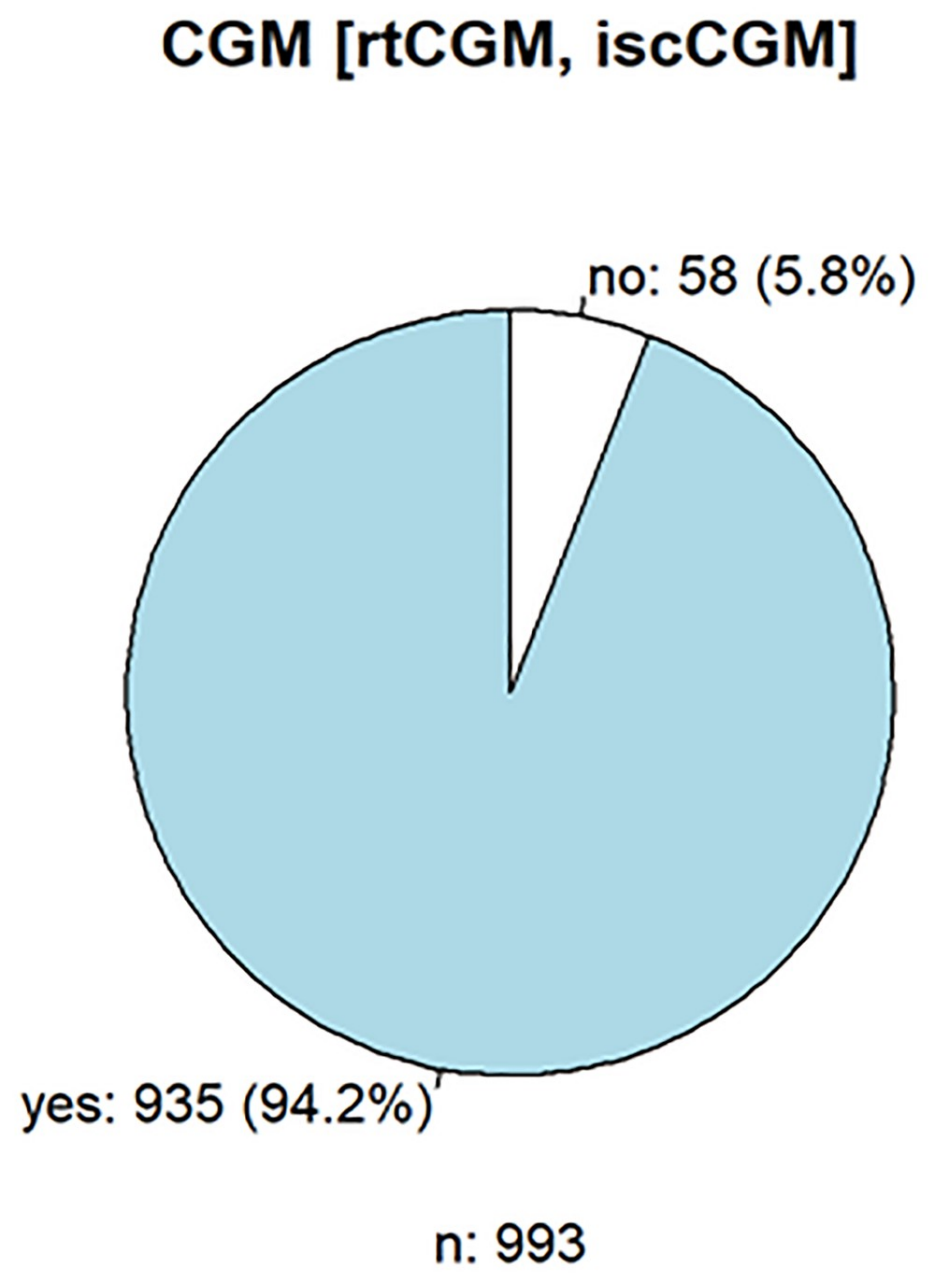
Frequency of continuous glucose monitoring used in diabetes therapy.

1054 persons responded, of whom seventy-six percent used an insulin pump, and 94% used a CGM (Figs 1 and 2), which shows that the acceptance and use of the current possible therapies for DMT1 are high.

Of 962 respondents, 731 (75.9%) used an insulin pump. Approximately 42% had the Medtronic MiniMed^™^ 640G insulin pump and 14% had the Insulet Omnipod and Accu-Check^®^ Spirit combo. A total of 101 patients (9.7%) used a DIYCL; the insulin pump DANA Diabecare^®^ R/ RS was used most frequently (55.0%) by these patients. The reason is the open interfaces of the pump, which enable the device to be integrated into the DIYCL via Bluetooth^™^. Overall, 66.0% of the users (n = 696) were satisfied with the insulin pumps, especially the operation (n = 129, 18.7%), the hypoglycemia switch-off (n = 108, 15.6%), the size or weight (n = 88, 12.7), possible CGM connection (n = 87,12.6), and tubeless function (n = 68, 9.8%). However, the size or weight (n = 107, 15.5%), the (missing) pump remote control/control (e.g., via app; n = 104, 15.0%), the menu navigation/operation (n = 76, 11.0%) and catheter/plaster/tube (n = 51, 7.4%), and the CGM connection/connectivity (n = 115, 16.6%) were mainly rated negatively. Although the respondents see a future in conventional insulin pump therapy with cannula and catheter tubing (n = 345, 50.3%), the patch insulin pump follows close behind (n = 307, 44.8%).

**Fig 3:**
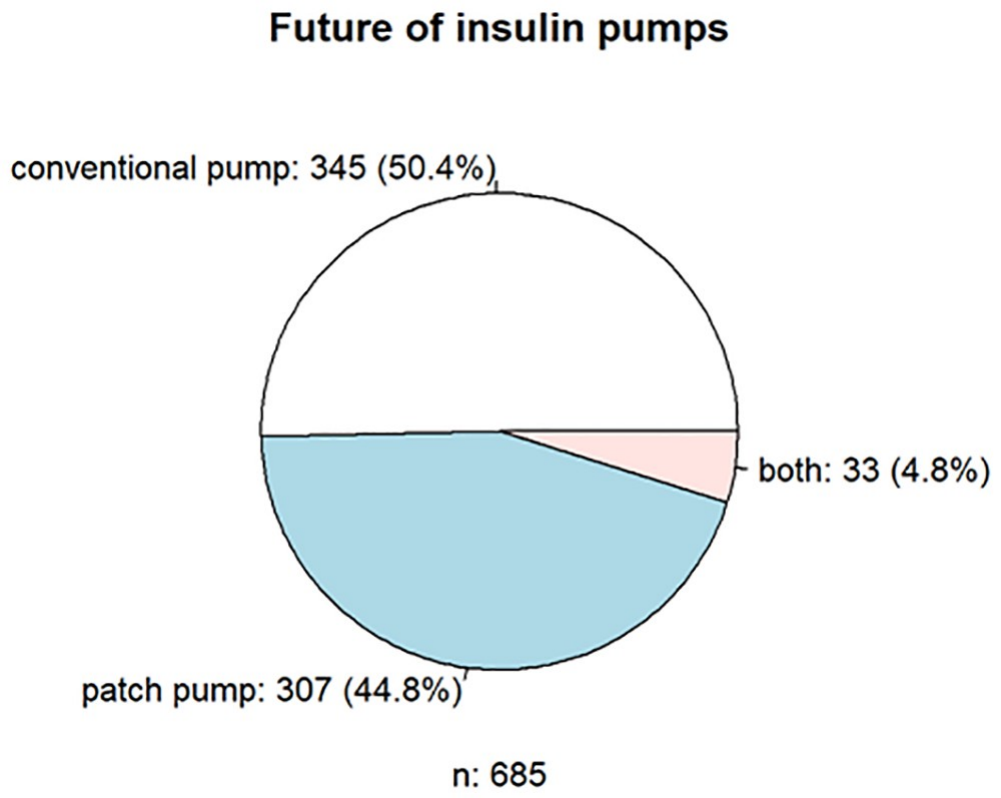
Respondents’ preferences regarding insulin pumps and CGM.

**Fig 4:**
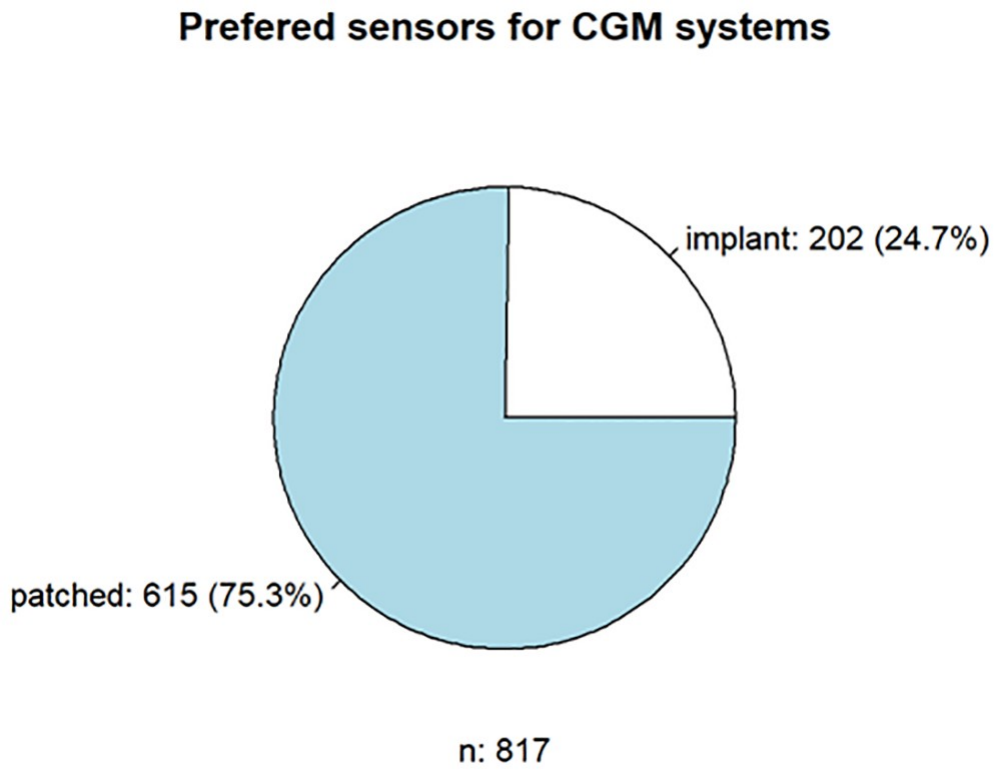
Respondents’ preferences regarding insulin pumps and CGM.

A total of 838 (79.5%) patients responded to the questionnaire on CGM. The most popular CGM was the FreeStyle Libre 1 from Abbott (n = 263, 31.4%), the Medtronic Enlite Sensor (n = 174, 20.7%), and the Dexcom G6 (n = 171, 20.4%). Notably, the FreeStyle Libre will be replaced in degrees by its successor model, the FreeStyle Libre 2 (n = 105, 12.5%). User satisfaction was 79.8% positive (n = 841). The main positive aspects were the flexibility of use of the CGM system (n = 177, 21.0%), the alarms for hypoglycemia or hyperglycemia (n = 155, 18.4%), the size or weight (n = 94, 11.2%), the accuracy (n = 87, 10.3%), the wearing time (n = 68, 8.0%), and the connection to the insulin pump (n = 66, 7.8%). Negative aspects were the wearing time of CGM (n = 121, 14.38%), (no) smartPhone/smartWatch readability (n = 107, 12.7%), accuracy (n = 104, 12.3%), adhesive tape (n = 101, 12.0%), size/weight (n = 87, 10.3%), and application/initialization of the sensor (n = 55, 6.5%). More than three-quarters (77.5%, n = 817) of the respondents tended to use subcutaneously patched sensors, in contrast to 25% (n = 202) of the respondents preferring implanted CGM systems (Figs 3 and 4). Regarding insulin pumps and CGM, some aspects were considered both positively and negatively due to the use of different models.

### DIYCL systems

In the DIYCL part of the survey, the respondents (n = 86) mostly used the AndroidAPS system (92%), followed by OpenAPS and Loop for iOS. This distribution roughly corresponds to the population of DIYCL system users in Germany.

**Fig 5:**
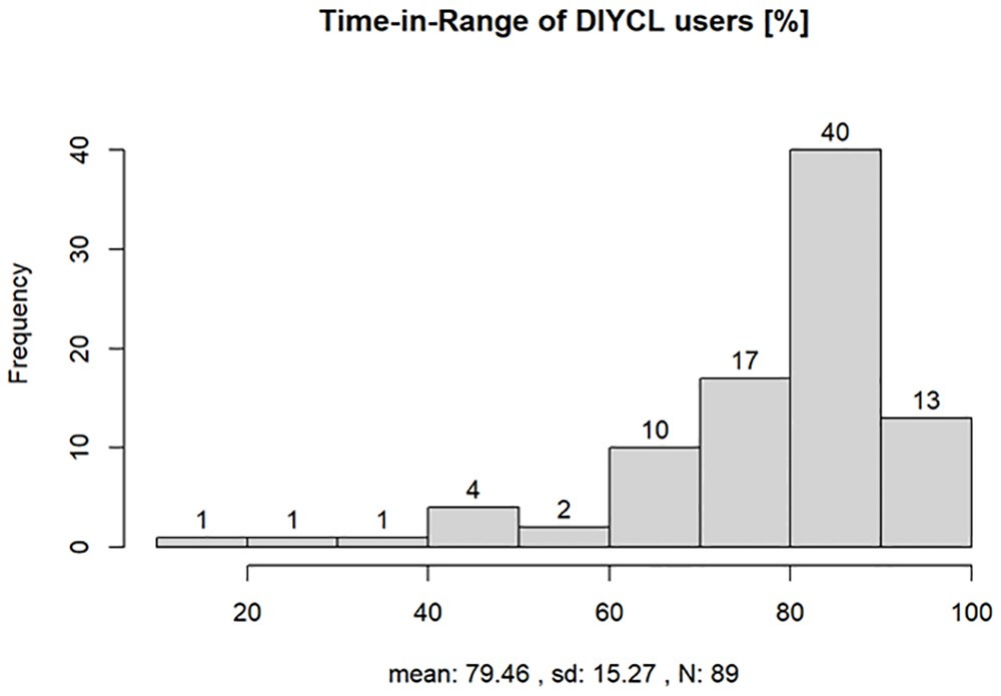
Time in range reported by users of a DIYCL system, mean range was 79,46%, SD 15.27, 89 patients responded.

In question 21 (see S1 Appendix), the survey participants using a DIYCL system were asked about their TIR. The mean value was 79.5% (n = 89). The following question asked about the improvement in HbA1c using the DIYCL system. Forty-seven of 89 (52.8%) participants stated that their HbA1c levels improved significantly (>1.0%) and 34 (38.2%) participants stated that their HbA1c levels improved a little (<1.0%). More than 93% of the respondents did not experience an increased rate of hypoglycemic episodes (Fig 5).

**Fig 6:**
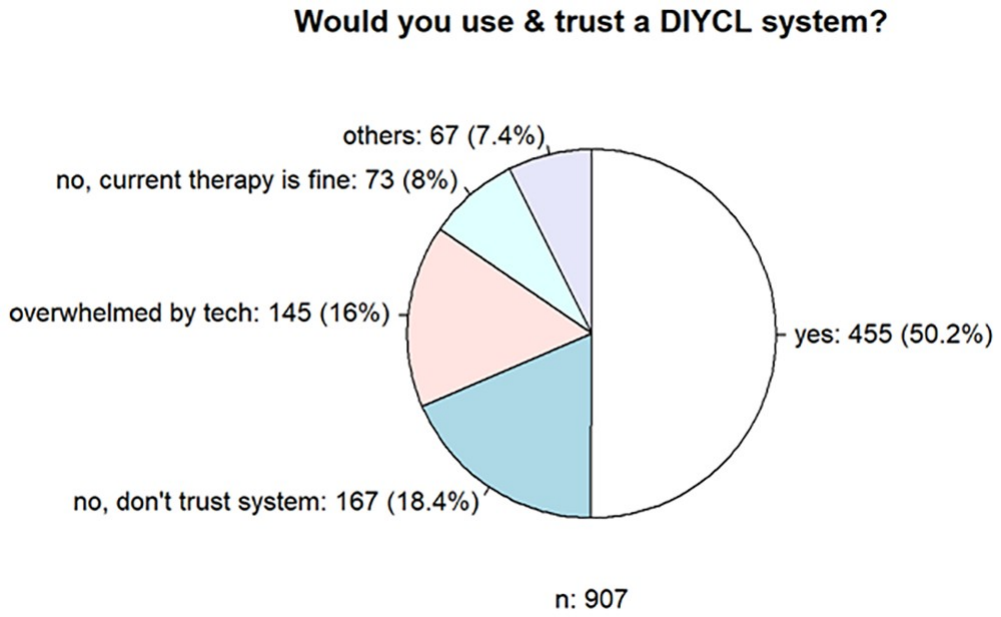
Assessment of the potential use and confidence in a DIYCL system.

When asked to evaluate DIYCL systems, the main positive aspects were better therapy quality/TIR (n = 39, 43.8%), better sleep quality/nightly safety (n = 20, 22.5%), automatic increase/decrease in insulin delivery depending on glucose levels (n = 17, 19.1%), fewer hyperglycemia episodes (n = 16, 17.9%), and better management of the disease (n = 9, 10.1%). The main criticisms were the complexity of the DIYCL system (n = 13, 14.6%), the documentation and instructions for the construction of the DIYCL system (n = 7, 7.8%), the extension of the system for the automatic detection of insulin sensitivity (n = 4, 4.5%), the lack of institutional approval (n = 4, 4.5%), a connection to the insulin pump that needs to be optimized (n = 3, 3.3%), insulin action (n = 3, 3.3%), visual presentation (n = 3, 3.3%), and the cumbersome updates for the DIYCL system (n = 3. 3.3%). The fifth part of the survey was aimed at future therapy. Question 26 evaluated the use of and trust in a DIYCL system. Of 907 respondents who answered the questions, 455 (50.2%) stated that they are confident about such a system and would use it, whereas 18.4% (n = 167) had no trust in the system, 15.9% (n = 145) had concerns about the technology, and 8.0% (n = 73) saw no need for improvement in their current therapy (Fig 6).

This relationship changed when asked about potential trust and application in a commercial CL system. Of the 902 respondents, 85.5% stated that they would trust such a system, whereas 8.0% (n = 84) would not, and another 8.0% saw no need for improvement in their current therapy. This finding is similar to previous surveys [25].

One question related to possible improvements. In particular, an automation of the therapy (n = 189, 17.9%) was mentioned. Furthermore, a smaller size or lower weight (n = 72, 6.8%), a connection of the products by a smartphone or smartwatch (n = 63, 5.8%), uniform interfaces or cooperation between manufacturers (n = 60, 5.7%), and simpler operation or usability (n = 45, 4.2%) were also mentioned. This question also indicated that patients have a positive attitude towards digital and automated therapy.

### Statistical analysis

A correlation analysis of HbA1c and TIR was performed as follows: First, the two results from the survey were tested for normality using the Shapiro-Wilk test, finding that the two variables in the survey results are not normally distributed (p<0.001). Spearman rank correlation analysis resulted in a correlation coefficient of -0.54 (p<0.001); thus, with increasing TIR, the HbA1c value decreases. This value can be interpreted as being of moderate effect strength. Similar effect strengths were reported by other studies [26, 27].

**Fig 7:**
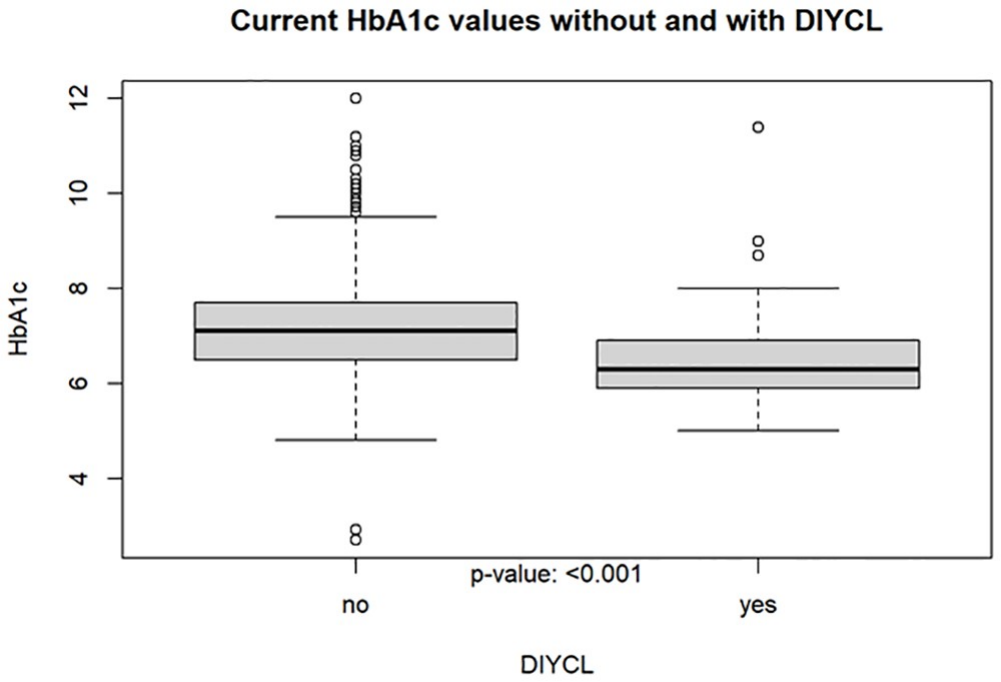
HbA1c levels in patients with and without a DIYCL system, HbA1c was significantly better in the DIYCL group, p-value <0.001.

**Fig 8:**
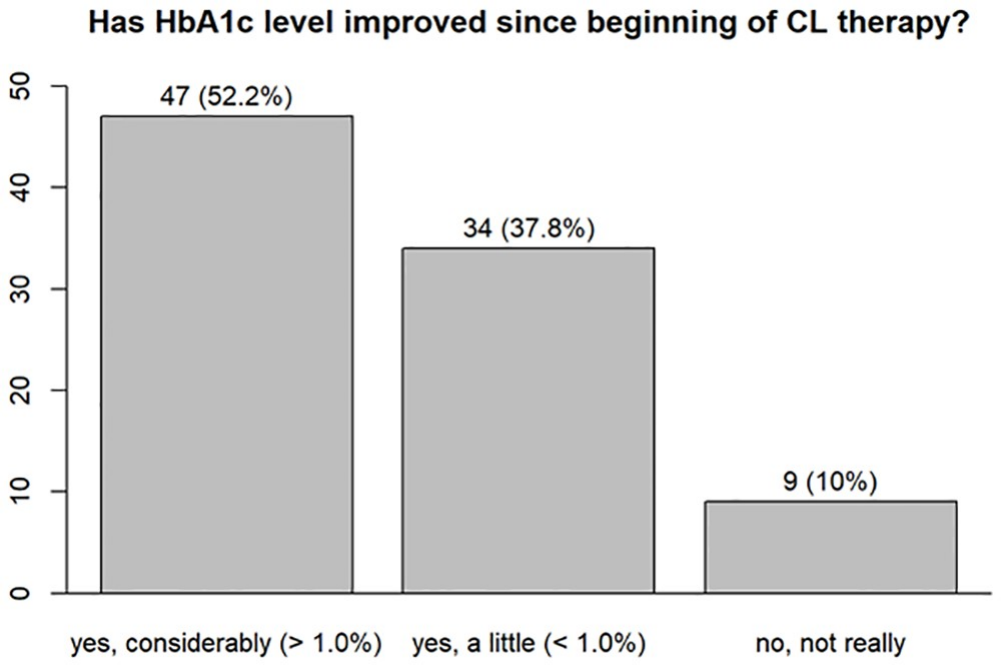
Improvement in HbA1c.

We also examined whether users of a DIYCL system had a lower HbA1c value in the survey (Figs 7 and 8). The t-test showed that the HbA1c values were significantly better among users of DIYCL systems than patients who do not use such a system (p<0.001).

This result confirms the need for the commercial development of a CL system, as it provides better long-term glucose control, minimizing the short- and long-term complications of diabetes.

The additional customer benefit analysis was intended to show which requirements are important for products related to the insulin pump and CGM. The most popular insulin pumps and CGM devices were ranked according to customer benefit and the three products with the highest level of user satisfaction selected. To ensure representativeness, the devices had to be rated by at least 3% of the respondents. The criteria for assessing the additional benefit of an insulin pump were size, operability, display, connectivity, and catheter placement, and the additional benefit of a CGM system was assessed by size, operability, data display, connectivity, mounting of sensor and transmitter, and CGM wearing time.

**Fig 9:**
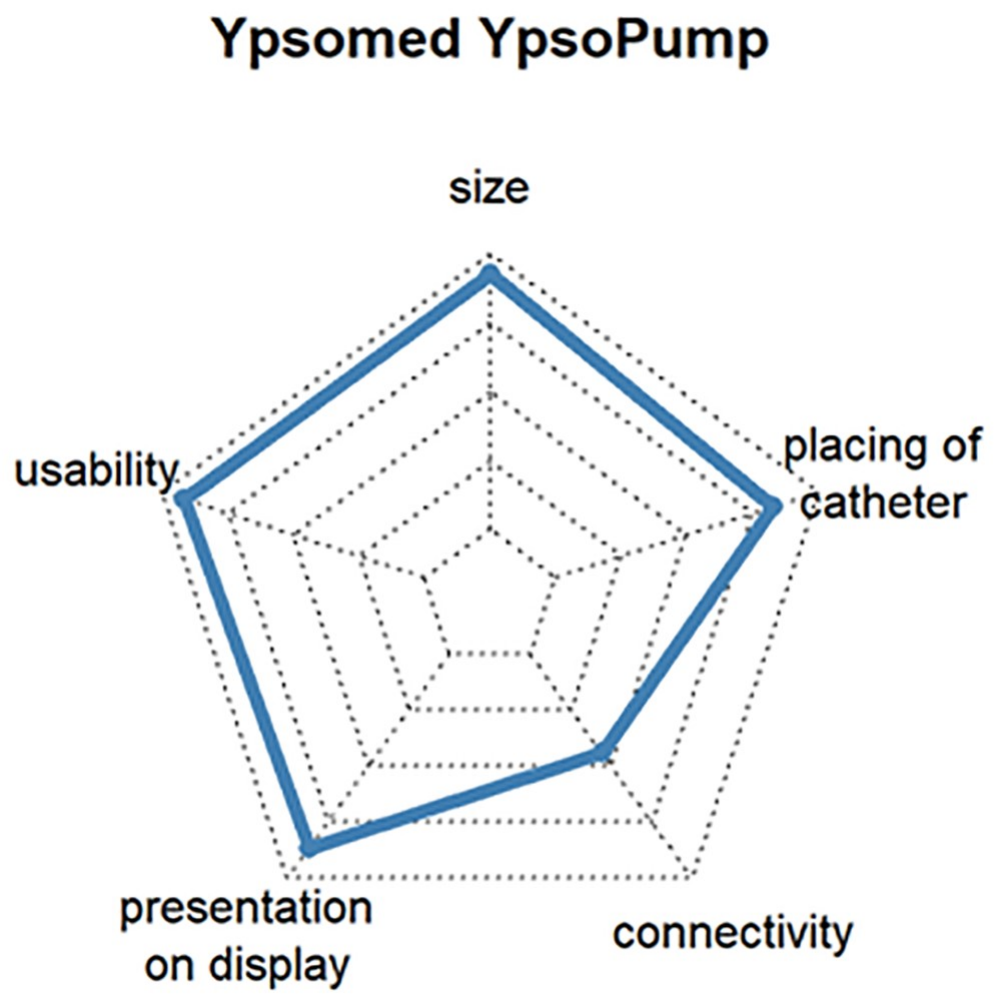
Customer satisfaction with the most popular insulin pumps by size, catheter handling, connectivity, presentation on display and usability.

**Fig 10:**
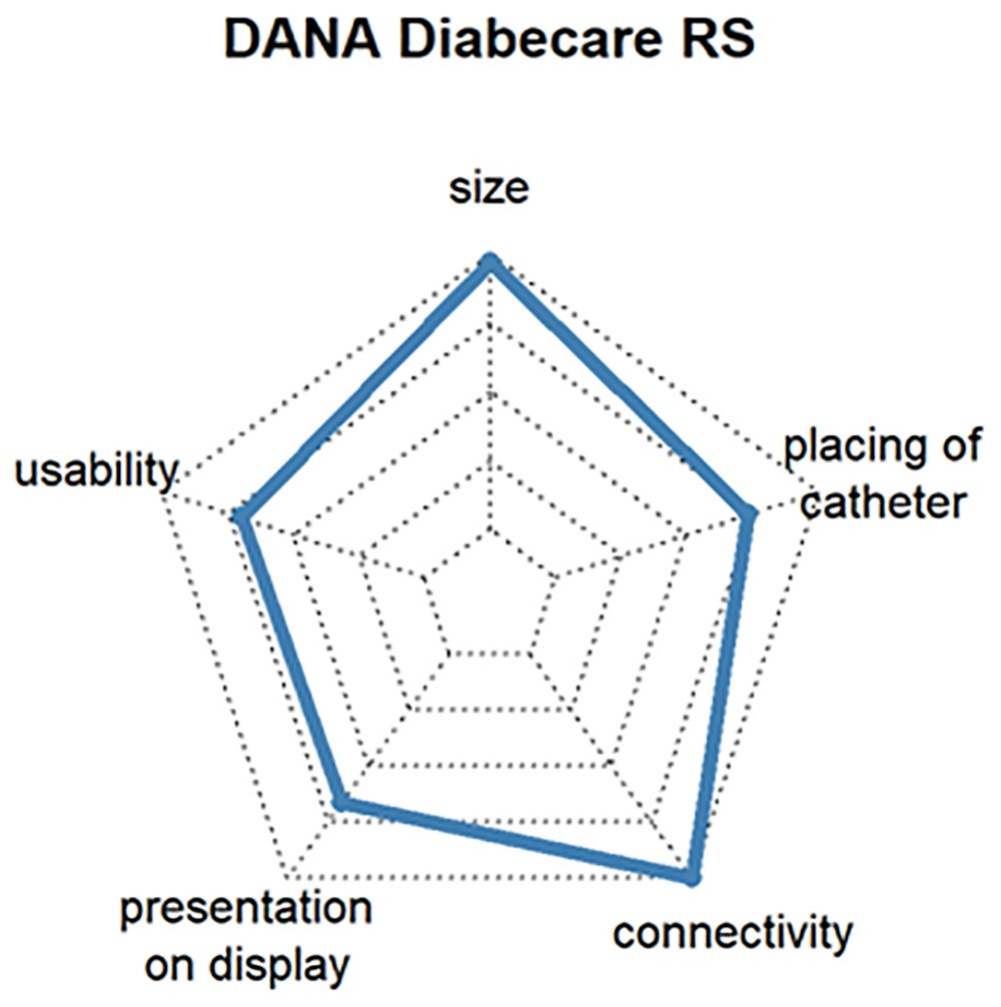
Customer satisfaction with the most popular insulin pumps by size, catheter handling, connectivity, presentation on display and usability.

**Fig 11:**
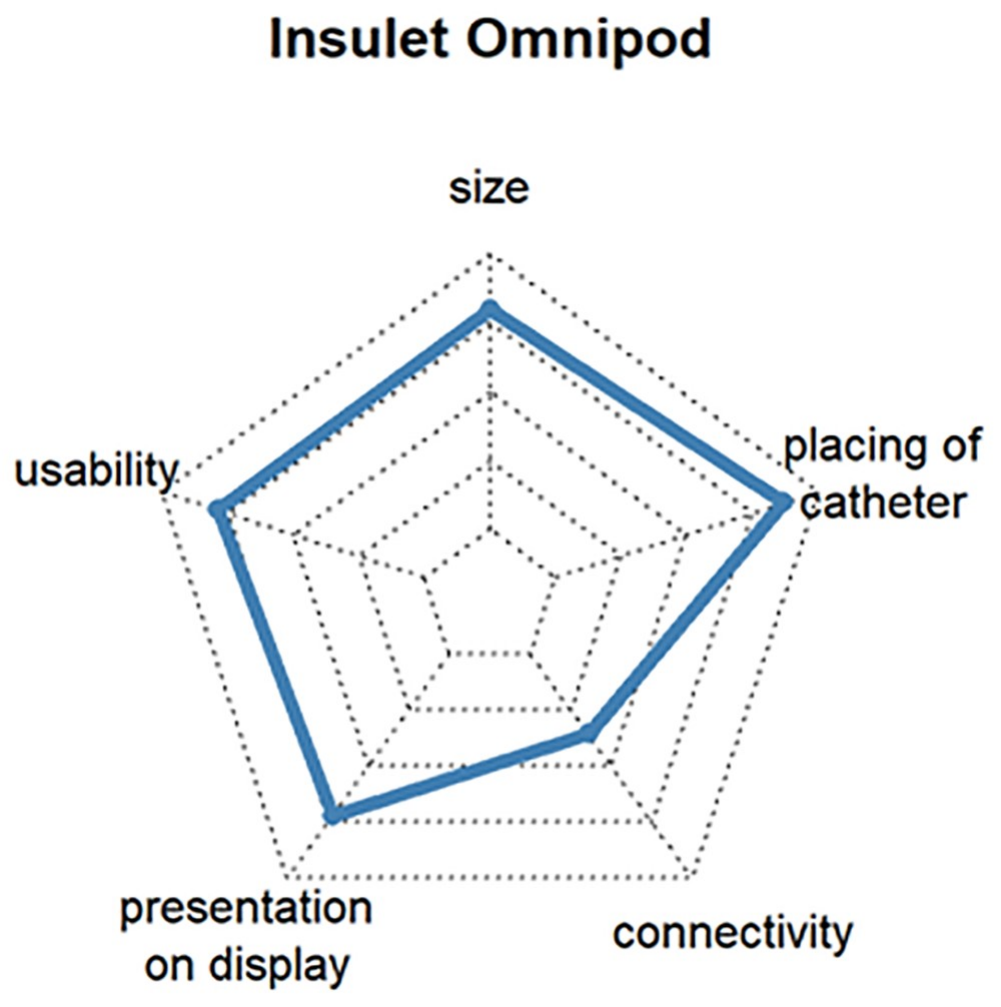
Customer satisfaction with the most popular insulin pumps by size, catheter handling, connectivity, presentation on display and usability.

Questions 7 and 9 of the survey were used to select the three insulin pumps with the highest customer satisfaction. The mean satisfaction values for all insulin pumps varied between 68% and 88%. The following three insulin pumps turned out to be the most popular:

- Ypsomed YpsoPump, which is the smallest conventional pump, can be operated by touch screen and might be connective with Dexcom G6 in 2021 (satisfaction: 86%, respondents: 3.3%). Fig 9
- DANA Diabecare^®^ RS, which has good connectivity with other assist devices and is operated by smartphone via Bluetooth (satisfaction: 80%, respondents: 6.3%) Fig 10
- Insulet Omnipod^®^, which is the first tubeless patch insulin pump on the German market. The insulin pump is located directly on the cannula and is approved for water contact, such as with swimming (satisfaction: 77%, respondents: 11.3%). Fig 11

**Fig 12:**
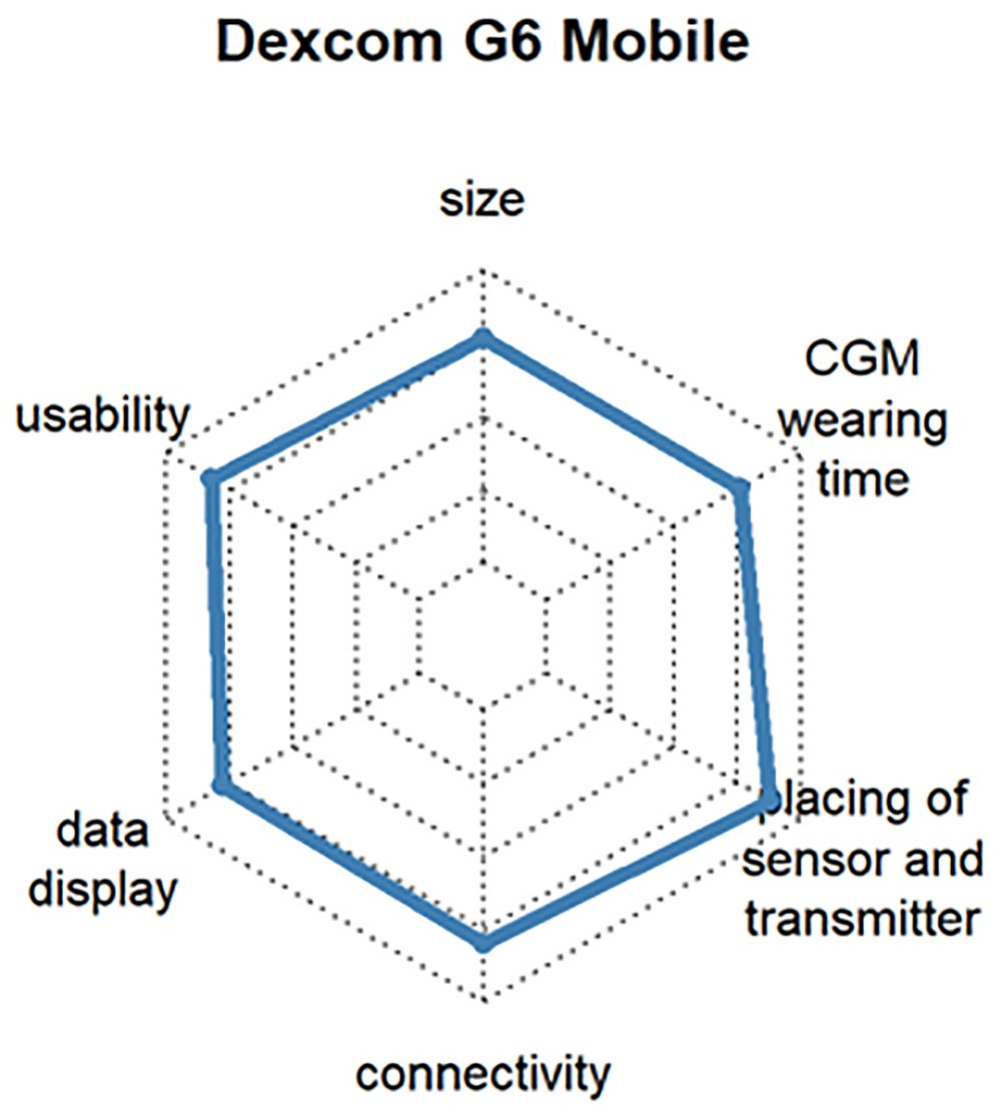
Customer satisfaction with the most popular CGM systems regarding size, usability, wearing time, display, connectivity and handling.

**Fig 13:**
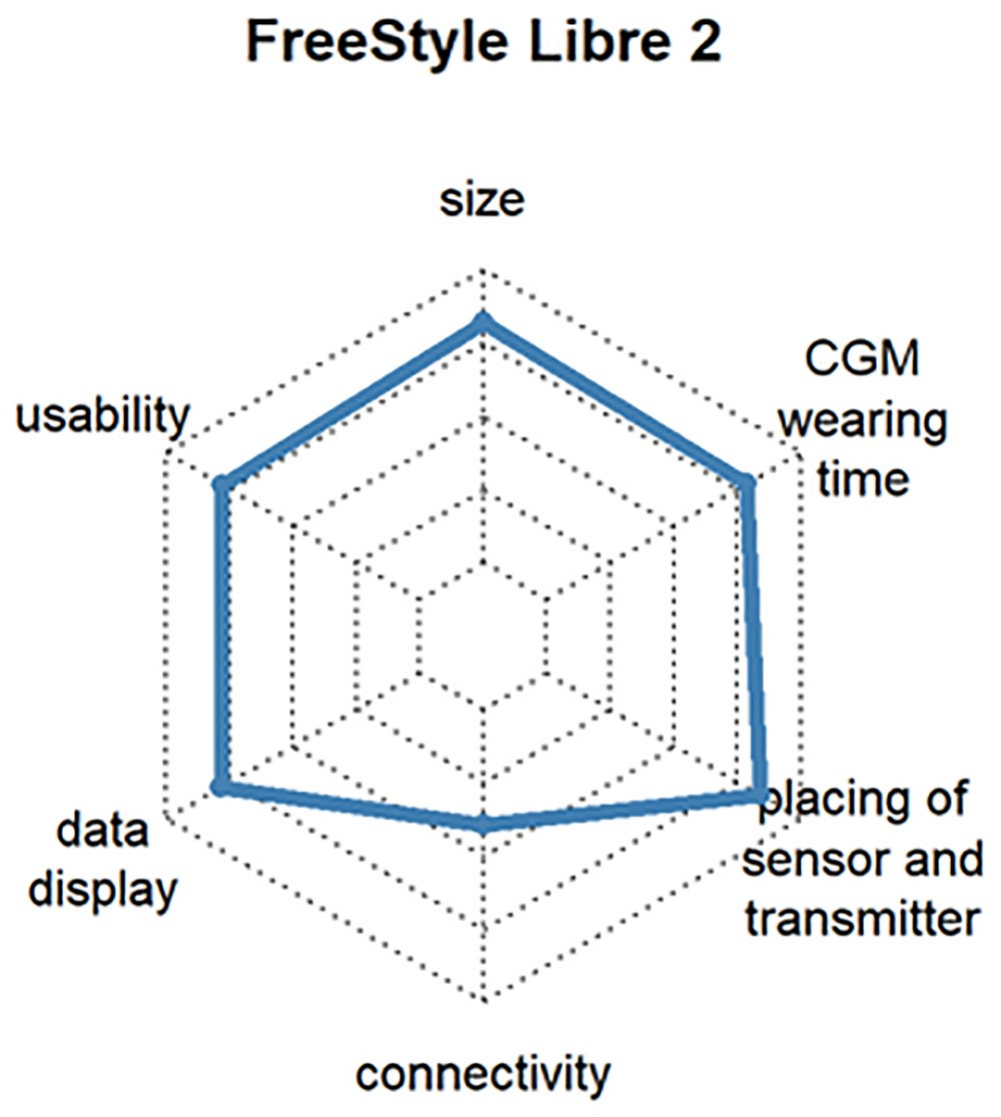
Customer satisfaction with the most popular CGM systems regarding size, usability, wearing time, display, connectivity and handling.

**Fig 14:**
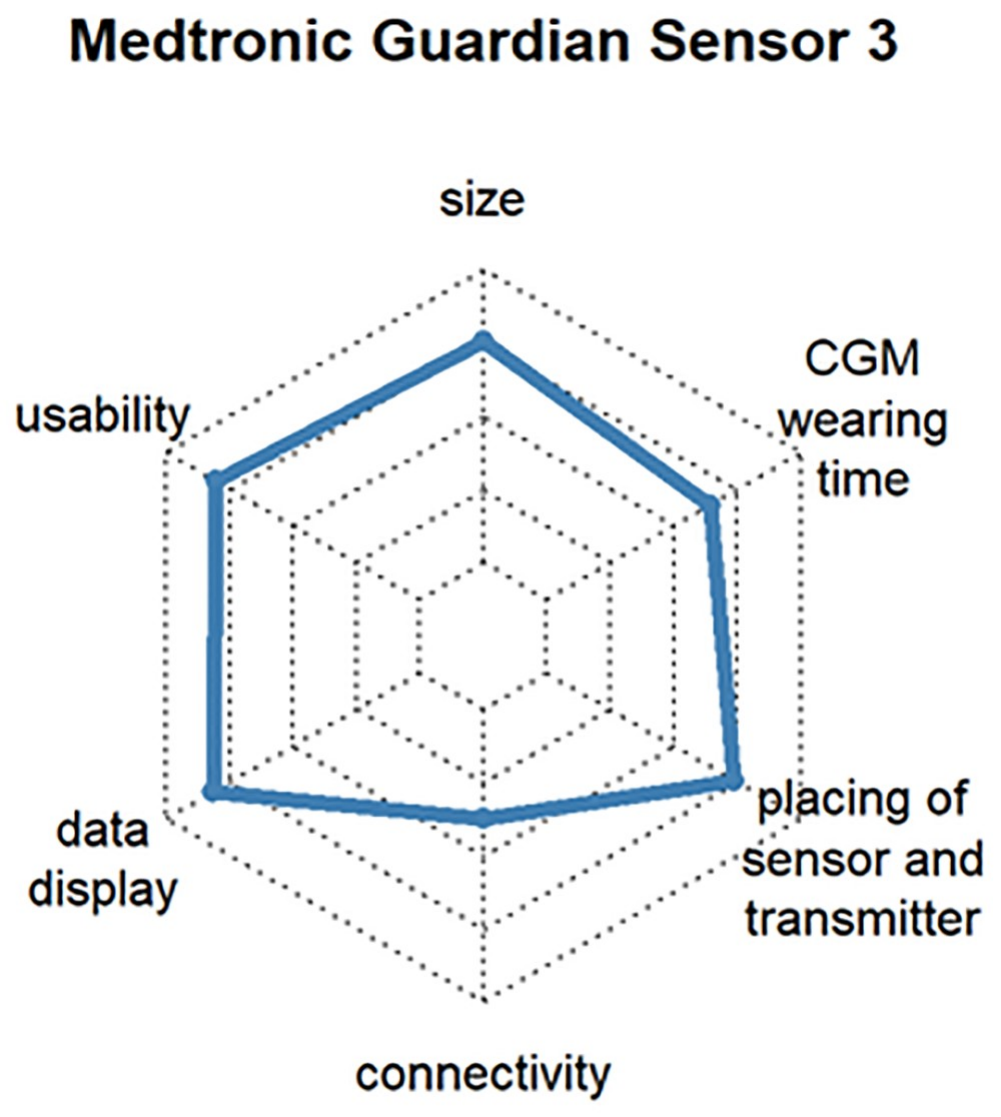
Customer satisfaction with the most popular CGM systems regarding size, usability, wearing time, display, connectivity and handling.

Questions 13 and 16 evaluated the customer satisfaction with CGM systems. The mean satisfaction values for all CGM systems varied between 63% and 84%. The latest version of CGM systems from the same manufacturer was chosen. The older versions are also given in square brackets below. Thus, the following selection was made:

- Dexcom^®^ G6, which determines the blood glucose value every 5 minutes and transmits it via Bluetooth^™^ to many end devices. The system also extrapolates the glucose curve over 20 minutes into the future and issues a warning if hypoglycemia or hyperglycemia is expected (satisfaction: 82%, respondents: 16.9%) [User of Dexcom^®^ G5 Mobile (satisfaction: 84%, respondents: 6.6%)]. See Fig 12
- Abbott FreeStyle Libre 2, which has optional alarms for hypoglycemia or hyperglycemia and trend arrows. It is small compared to other CGM systems. At the time of the survey, the system could not yet be evaluated with a smartphone, but this was changed soon after (satisfaction: 73%, respondents: 10.6%) [User of Abbott FreeStyle Libre 1 (satisfaction: 75%, respondents: 27.0%)]. See Fig 13
- Medtronic Guardian^™^ Sensor 3, which works in combination with Medtronic insulin pumps (e.g., Medtronic 640G, Medtronic 670G, etc.). In the case of hypoglycemia, the combination with an insulin pump can stop basal delivery, making Medtronic the first company to offer a commercial near-closed-loop system (satisfaction: 71%, respondents: 3.6%) [User of Medtronic Enlite^™^ Sensor (satisfaction: 69%, respondents: 17.5%)]. See Fig 14

Overall, the average user satisfaction with insulin pumps and CGM systems across all devices was approximately 75%. The identified TIR for CGM systems was approximately 66%. However, users of DIYCL systems had a higher average TIR of 79.5% and a significantly lower HbA1c value.

## Discussion

We found that most patients have great interest in the further development of easy to handle therapeutic devices, which explains the large number of participants in our survey. Over 50% of all respondents would use a commercial full CL system if available. Whether the prevalence of DIYCL users compared to non-users in our survey corresponds to prior data must be assessed critically [25] Nevertheless, the result is positive, because a greater power was achieved with the DIYCL survey.

Automated insulin delivery and CL systems were shown to provide a significant improvement in HbA1c values, a significant reduction in hypoglycemia and hyperglycemia or increased TIR, and an improvement in the quality of life and sleep [28–30]. Lewis et al. achieved similar results in a retrospective crossover analysis of 20 participants for measurements 4 to 6 weeks before using the OpenAPS system and after 4 to 6 weeks of using the system [31]. In our survey, 46 of 58 DIYCL users reported better sleep quality/nightly safety, fewer interactions, and better management of the disease. A multicenter trial with 20 very young children between 1 and 7 years of age using an experimental closed loop system containing a modified Medtronic^™^ 640G insulin pump (investigational use only; Medtronic, Northridge, California), a real‐time continuous glucose sensor (Enlite 3, Medtronic), and the FlorenceM CL system control algorithm version 0.3.46 reported similar results. Responders especially emphasized the improvement in quality of sleep with CL for both caregivers and users [32]. We could reproduce these findings.

Only about half of the non-DIYCL users in our survey would trust a self-constructed system. This relationship changed when asked about potential trust in a commercial CL system, and more than 75% of the respondents stated that they would potentially use such a system.

However, despite the apparent need for more automation in diabetes therapy, only the HCL Medtronic Minimed^™^ 670G, as the world’s first insulin pump with adaptive basal insulin delivery, has become marketable thus far, and eligible for reimbursement in Germany in September 2019 [33]. One possible reason could be the high bureaucratic requirements for the approval and reimbursement of medical devices [34].

Unannounced carbohydrate intake and meal-associated insulin delivery still require a pragmatic approach and patient intervention. Due to the progress in the development of hardware and software for smartphones, machine learning and image processing algorithms could offer the possibility of calculating the amount of carbohydrates using photos or videos. For this purpose, the food is automatically identified, grouped, segmented, and quantified as shown on the image, and feedback is provided to the insulin pump [35].

Another problem with CL systems is safety concerns, as all three major components could potentially fail. Errors can result from insufficient calibration, slow sensing, miscommunication between the components, or human error. Legally, the question arises as to who is liable in the case of a medical emergency due to the DIYCL system, or if a third party suffers damage due to a fault in the system. Thus far, only one medical incident has been identified in which a patient using a DIYCL system suffered hypoglycemia and needed medical attention. However, the patient recovered quickly and suffered no consequential damages [36]. As a result, the U.S. Food and Drug Administration (FDA) issued a warning about the use of unapproved medical devices [37]. According to the current state of affairs (research of 09.08.2019), no official decisions on the situation have been made. Nevertheless, the product liability will likely expire due to improper use of the medical devices and pass completely to the patient, such as in the case of accidents [38].

Furthermore, the many different types of events that can lead to fatality can be difficult to calculate. As several devices may be required for the implementation (e.g., insulin pump, CGM, smartphone, etc.), they must be ensured to be within range to communicate with each other. Therefore, the number of devices should be minimized as much as possible [39]. The initialization of the system should also contain as few manual actions as possible, and the calibration of the CGM could be completely eliminated, as in the Abbott Free-Style Libre 2 [40].

Another promising approach seems to be the use of multi-hormonal systems consisting of insulin and glucagon. Data have shown better control of serum glucose levels and fewer hypoglycemic events, especially at night, compared to insulin pump only, but no significant benefit has been shown compared to single hormone systems [41]. Additional benefits of the dual-hormone artificial pancreas were reduced risk of hypoglycemic events during exercise and prevention of late-onset post-exercise hypoglycemia [42]. However, currently, the availability of a stable liquid dosage form of glucagon is limited, and glucagon has to be changed daily in multi-hormonal systems [43, 44].

## Conclusion

Our survey showed that the interest in automated systems for the treatment of diabetes mellitus exceeds previous use. Although it has been possible for a young, tech-savvy community to produce and use CL systems with very good results, broad commercial application has failed to materialize. Starting from the previous CL approach, we must work iteratively towards a fully automated CL system. Initiatives such as the DIYCL system can also help. In the coming years, more 2nd generation CL systems are expected to enter the market according to JDRF classification. Based on the experience with these systems and with further research in, for example, multihormonal approaches and the integration of additional sensors, the marketability of a fully automated CL system would be possible. This would make it feasible to minimize secondary diseases in patients with DMT1 and improve the quality of life of those affected.

## Supporting information

S1 Appendix (DOCX)
