# Supplementary material for: Survey about do-it-yourself closed loop systems in the treatment of diabetes in Germany
Source: PLoS One. 2020 Dec 17;15(12):e0243465. doi: 10.1371/journal.pone.0243465 (PMC7746287; doi:10.1371/journal.pone.0243465)
Supplement: S1 Appendix — (DOCX) [file pone.0243465.s001.docx]

| Introduction | | |
| --- | --- | --- |
| 1. | Who is diagnosed with diabetes mellitus? | Myself (n=776), My child (266), My partner (10), My parent (3), [free text field] |
| 2. | What type of diabetes do you have? | Type 1, Type 2, Type 3c, Gestational diabetes, Other form |
| 3. | What's your current HbA1c level? | [free text field in %] |
| 4. | How long have you been diagnosed diabetes mellitus? | 0-2 years, 2-5 years, 5-10 years, 10-20 years, 20-30 years, 30-40 years, 50-60 years, > 60 years |
| 5. | What tools do you use for your diabetes management? | Insulin pump (737), insulin injection (299), blood glucose meter (495), CGM (rtCGM, iscCGM) (959), [free text field] |
| 6. | Do you use a do-it-yourself closed loop (e.g. OpenAPS)? | Yes, No |
| Questions to current therapy forms: Insulin pump | | |
| 7. | Which insulin pump do you use? | Accu-Chek Insight  Accu-Chek Spirit Combo  Animas Vibe  DANA Diabecare RS  Insulet Omnipod  Medtrum A6 Touch Care  MiniMed Veo 554/754  MiniMed 640G  MiniMed 670G  YpsoPump  [free text field] |
| 8. | How satisfied are you with the insulin pump with regard to the following aspects: | Size, Operability, Presentation on screen, Connectivity to CGM, etc., Catheter placement  [Rating: --- (very unsatisfied), --, -, +, ++, +++ (very satisfied)] |
| 9. | How satisfied are you with the insulin pump overall? | [Slider between 0% (very unsatisified) and 100% (very satisfied)] (423 <50%, 631 ≥50%) |
| 10. | Which facts do you find particularly good about the insulin pump? | [free text field] |
| 11. | Which facts do you find to be in need of improvement about the insulin pump? | [free text field] |
| 12. | Do you see the future more in a patch insulin pump or in a conventional insulin pump with cannula and catheter tube?  (Explanation: A patch insulin pump has an integrated cannula and has no catheter tubing.) | Conventional insulin pump with cannula and catheter tubing (380)  Patch insulin pump (343) |
| Questions to current therapy forms: CGM | | |
| 13. | Which CGM system do you use? | Medtronic Enlite Sensor  Medtronic Guardian Sensor 3  Dexcom G4 Platinum  Dexcom G5 Mobile  Dexcom G6  Medtrum A6 Touch Care CGM  FreeStyle Libre 1  FreeStyle Libre 2  EverSense / Eversense XL  [free text field] |
| 14. | What is your time-in-range? ( What percentage of values are within your defined glucose target range? If you 1do not have exact values, please estimate.) | [free text field in %] |
| 15. | How satisfied are you with the CGM system in the following aspects: | Size, Usability, Presentation on screen, Connectivity to Smartphone, etc., Attaching the CGM sensor and transmitter, Wearing time  [Rating: --- (very unsatisfied), --, -, +, ++, +++ (very satisfied)] (273 <50%, 781 ≥50% |
| 16. | How satisfied are you with the CGM system overall? | [Slider between 0% (very unsatisfied) and 100% (very satisfied)] (355 <50%, >≥695) |
| 17. | Which facts do you find particularly good about the CGM system? | [free text field] |
| 18. | Which facts do you find to be in need of improvement about the CGM-System? | [free text field] |
| 19. | Which sensor shapes for a CGM system would you prefer? | Implanted (628)  Patched (209) |
| Questions to Do-It-Yourself (DIY) Closed Loop | | |
| 20. | Which closed loop system do you use? | Open APS (3)  Loop (4)  Android APS (79)  [free text field] |
| 21. | What is your time-in-range?  (What percentage of values are within your defined glucose target range? If you do not have exact values, please estimate.) | [free text field in %]  (11 <70%, 75 ≥70%) |
| 22. | Has your HbA1c value improved since starting therapy with the closed loop system? | Yes, clearly (>1,0%).  Yes, a little bit (<1,0%),  No |
| 23. | Have you had more hypoglycemia since the start of therapy with the closed loop system? | Yes  No |
| 24. | Which facts do you find particularly good about the closed loop system? | [free text field] |
| 25. | Which facts do you find in need of improvement about the closed loop system? | [free text field] |
| Questions to future therapy forms | | |
| 26. | Would you use and trust a self-built closed loop system? (This means that an insulin pump regulates the insulin delivery based on measured glucose values and algorithms.) | Yes  No, I am satisfied with my current therapy.  No, I am overchallenged with the technology.  No, I do not trust the system.  [free text field] |
| 27. | Would you use and trust a commercial closed loop system? (This means that an insulin pump regulates the insulin delivery based on measured glucose values and algorithms.) | Yes  No, I am satisfied with my current therapy.  No, I am overchallenged with the technology.  No, I do not trust the system.  [free text field] |
| 28. | What improvements do you want for future diabetes therapy products? | [free text field] |
| Demographic questions | | |
| 29. | What age group do you belong to? | 0-9 years, 10-19 years, 20-29 years, 30-39 years, 40-49 years, 50-59 years, 60-69 years, Older than 70 years |
| 30. | What is your gender? | Male, Female, Diverse |
| 31. | Which state do you come from? | No information (170), Berlin (30), Brandenburg (25), Saxony Anhalt (19),  Saxony (38), Thuringia (22), Mecklenburg Western Pomerania (13), Schleswig-Holstein (34), Lower Saxony (91), Hamburg (18), Bremen (5), Hessen (85), Rhineland Palatinate (41), Baden Wurttemberg (107), Saarland (11), Bavaria (140), North Rhine Westphalia (205) |
